# Child Malnutrition in Gaza Requires Adequate Diets and Continuity of Care

**DOI:** 10.1111/mcn.70292

**Published:** 2026-11-14

**Authors:** Abdishakur Ahmed Mahad, Abdisamad Abdihakim Aadam, Zakia Abdalla Rashiid, Mohamed Mahdi Hashi, Abdimalik Dahir Ali, Ilyas Abdullahi Khalif

**Affiliations:** ^1^ ‎Faculty of Health Sciences and Tropical Medicine Somali National University ‎Mogadishu, ‎Somalia; ^2^ Benaadir Research, Consultancy & Evaluation Center Mogadishu Somalia; ^3^ Center for Health Research and Innovation Somali National University Mogadishu Somalia

**Keywords:** acute malnutrition, child malnutrition, conflict settings, dietary diversity, food insecurity, Gaza, humanitarian crisis

## Abstract

Gaza's child malnutrition crisis reflects insufficient food availability, poor dietary quality, micronutrient deficiencies, disrupted treatment, displacement, insecurity, and constrained humanitarian access. Evidence before and during the 2023–2024 escalation documents household food insecurity and inadequate dietary diversity, alongside childhood anaemia. Conflict‐period studies report acute malnutrition among children younger than five, with geographic disparities and higher severe acute malnutrition in northern Gaza. Programme evidence indicates that outpatient nutrition care can improve anthropometric measures among children retained in treatment, while attrition linked to displacement and insecurity undermines continuity of care. This letter synthesises published studies and public reporting to argue that emergency responses must address immediate wasting and the conditions required for child health and development. We recommend integrated surveillance of dietary diversity, micronutrient status, geographic inequities, and treatment retention; coordinated delivery of therapeutic nutrition, infant and young child feeding support, immunisation, psychosocial stimulation, and water, sanitation, and hygiene services; and sustained humanitarian access with fuel and decentralised care. Recovery should be assessed not only by food deliveries or short‐term survival, but also by access to nutritious diets and essential child health services.

1

Globally, child malnutrition in conflict settings is often framed as a problem of calories alone. In Gaza, that framing does not fully capture children's nutritional and healthcare needs. The child health emergency persists because food availability alone does not establish nutritional adequacy, continuity of care, or safe humanitarian access. This distinction is relevant to fragile settings where famine prevention metrics may not fully capture children's nutritional and developmental needs (Horino et al. 2024; Rabayaa and Rabaya 2026).

Recent data show both the scale and complexity of the crisis. Before the 2023–24 escalation, 42.6% of surveyed refugee households with first‐grade children in Gaza were already severely food insecure, 68.9% of children failed to consume a minimally diverse diet daily, and 29.8% had anaemia, despite widespread food assistance coverage (Horino et al. 2024). During the conflict, nutritional status deteriorated sharply. In one cross‐sectional study of 1200 children under 5 years, overall acute malnutrition reached 16.6%, including 6.7% severe acute malnutrition and 9.7% moderate acute malnutrition, with the highest odds concentrated in North Gaza (Albelbeisi et al. 2024). A larger humanitarian clinic analysis of 22,901 children reported acute malnutrition in 8.92% overall, but severe acute malnutrition in North Gaza/Gaza City was 15.7% versus 0.4% in the south and middle areas (Hassneiah et al. 2025).

Available evidence also highlights constraints on response capacity. UNICEF‐linked reporting described more than 7000 children under 5 admitted to recovery programmes for acute malnutrition within 2 weeks in August, with over 15,000 cases expected that month, more than seven times the number reported in February (López‐Gil 2025). A prospective programme evaluation from Gaza City found gains in MUAC and weight among children retained in WHO‐standard outpatient care, yet reported 41.2% attrition, largely linked to displacement and insecurity (Helu et al. 2026). These findings highlight barriers to maintaining access and continuity of nutrition care, despite observed improvements among children retained in treatment (López‐Gil 2025; Helu et al. 2026).

This crisis warrants sustained scientific and policy attention. The available evidence raises concern about potential longer‐term developmental consequences, but does not establish their magnitude or predict specific educational or economic outcomes among children in Gaza. Emerging analyses further argue that a ceasefire, although essential, is insufficient if humanitarian access remains restricted and nutritionally appropriate supplies, water, sanitation, fuel, and maternal‐child services are not restored at scale (Rabayaa and Rabaya 2026; Hassneiah et al. 2025). Assessments of recovery should therefore consider nutritional and developmental needs alongside child survival. This letter draws on published evidence and publicly available reporting; the authors have no institutional affiliation or operational collaborator in Gaza and do not offer firsthand observations of the response.

Three actions are now justified. First, surveillance must move beyond crude food availability to track diet diversity, micronutrient status, treatment retention, and geographic inequities in children under 5 (Horino et al. 2024; Helu et al. 2026). Second, nutrition programming should integrate ready‐to‐use therapeutic foods, infant and young child feeding support, immunisation, psychosocial stimulation, and WASH rather than treating wasting in isolation (Hassneiah et al. 2025; López‐Gil 2025). Third, international agencies and donors should prioritise protected humanitarian corridors, fuel, and decentralised service models that can function during displacement (Rabayaa and Rabaya 2026; Hassneiah et al. 2025).

Assessing Gaza's child health recovery requires attention to adequate and diverse diets, sustained therapeutic and preventive care, and the conditions required for healthy development, including water, sanitation, fuel, and uninterrupted humanitarian access. Without these essentials, children may remain at risk of preventable illness and potential longer‐term developmental harm. Recovery should therefore be assessed through sustained access to adequate diets, nutrition care, and essential child health services.

## Author Contributions

A.A.M. conceptualised and designed the study, and drafted and finalised the manuscript. A.A.A., Z.A.R., M.M.H., A.D.A., contributed to the literature review. I.A.K. critically reviewed and supervised the manuscript and provided substantial intellectual input. All authors read and approved the final version of the manuscript.

## Funding

The authors have nothing to report.

## Conflicts of Interest

The authors declare no conflicts of interest.
